# Direct Dispersion of Graphene Oxide in Epoxy: Influence Of Processing Route on Thermal, Dynamic-Mechanical, and Impact Properties

**DOI:** 10.1021/acsomega.6c05607

**Published:** 2026-09-22

**Authors:** Fillip C. Alves, Carlos E. Moraes, Lucas C. da Silva, Luis F. P. Santos, Heitor L. O. Junior, Newton S. da Silva, Michelle L. Costa, Edson C. Botelho

**Affiliations:** † Materials and Technology Department, School of Engineering and Sciences, São Paulo State University (UNESP), Avenida Dr. Ariberto Pereira da Cunha, 333, Pedregulho, 12516-410 Guaratinguetá, São Paulo, Brazil; ‡ Lightweight Structures Laboratory (LEL), Institute for Technological Research of the State of São Paulo (IPT), Estrada Dr. Altino Bondesan, 500, Eugênio de Melo, 12247-016 São José dos Campos, São Paulo, Brazil; § Department of Biosciences and Oral Diagnosis, Institute of Science and Technology, São Paulo State University (UNESP), Avenida Engenheiro Francisco José Longo, 777, Jardim São Dimas, 12245-000 São José dos Campos, São Paulo, Brazil; ∥ Forza Composites, Avenida Shishima Hifumi, 2911, Urbanova, 12244-000 São José dos Campos, São Paulo, Brazil

## Abstract

Epoxy matrix nanocomposites reinforced with graphene oxide (GO) have attracted significant interest mainly due to the combination of the nanofiller’s exceptional properties with the versatility of epoxy resin, widely used in the aerospace, automotive, sports, and electronics applications. Achieving a homogeneous dispersion of the nanofiller within the polymer matrix remains a crucial challenge, as agglomerates can compromise the material performance. This study comparatively investigates the incorporation of 0.5 wt % GO using different direct dispersion methods into epoxy resin, including manual, mechanical, magnetic stirring, ultrasonic bath, and ultrasonic tip, without organic solvents. The techniques were evaluated in terms of dispersion efficiency and their effects on the mechanical, thermal, and dynamic-mechanical properties of the resulting nanocomposites. The results demonstrated that the used GO concentration enhanced the investigated properties across all dispersion methods. All nanocomposites exhibited an increase in storage modulus compared to pure epoxy resin, with NC4 (157%), NC3 (121%), and NC2 (107%) showing the most significant improvements, indicating enhanced interfacial interactions. Impact resistance was most significantly improved by dispersion via ultrasonic tip, while microscopy analyses (TEM and SEM) effectively characterized and distinguished the dispersion quality achieved by each technique. The ultrasonic tip method led to the most effective dispersion, resulting in a 70.6% increase in impact resistance and a 157% enhancement in storage modulus, evidencing its potential for high-performance nanocomposites.

## Graphical abstract

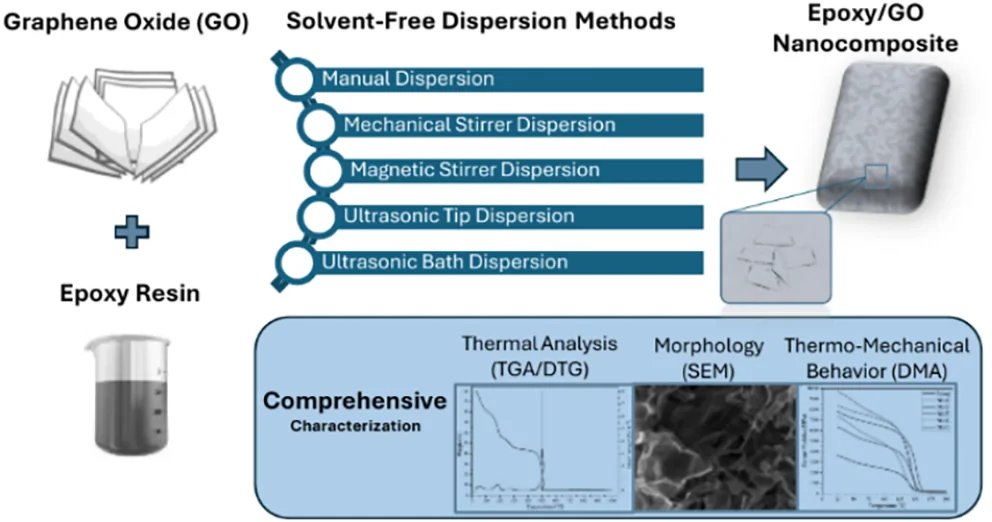

## 1 Introduction

Currently, polymer matrix composites are among the most prominent materials in several sectors, especially where lightweight applications are sought, such as in the aerospace, automotive, sports, and energy industries.^1–4^ The use of nanomaterials has contributed to the development and/or improvement of specific properties of composite materials, known as nanocomposites. They have attracted increasing attention in several fields mainly due to their ability to combine the unique properties of nanofillers with the advantages of polymer matrices.^5,6^

Epoxy resin, in particular, stands out as one of the most widely used matrices in the manufacture of nanocomposites due to its high mechanical strength, thermal stability, and versatility in industrial applications.^7,8^ These characteristics make the resin fundamental in fields such as aerospace, automotive, and electronics, where high-performance materials are essential.^9,10^

Among the most studied nanofillers, graphene has stood out due to its excellent mechanical properties, such as tensile strength (approximately 125 GPa) and its elastic modulus (around 1.1 TPa), making it about 100 times stronger than steel;^11^ its electrical and thermal conductivity (around 5 × 10^3^ W.(mK)^−1^), as well as its low density (0.77 mg.m^–2^).^12,13^ Graphene oxide (GO), in turn, presents chemical functionalities that facilitate interaction with the polymer matrix, which can improve dispersion and interfacial compatibility.^14,15^ Recent studies, as shown by Rehim (2022), have reported significant improvements in the electrical properties of epoxy resin composites with the use of graphene-based nanomaterials at a concentration of 1 wt %.^16^

One of the main advantages of using graphene oxide as a nanofiller is its ability to improve matrix properties, even at low concentrations.^17,18^ However, the homogeneous dispersion of nanofillers in the polymer matrix remains one of the biggest challenges in the development of nanocomposites. Poor dispersion can lead to the formation of agglomerates that compromise the efficiency of the nanofillers and the overall properties of the material.^19^ The effective dispersion of graphene oxide nanoparticles in polymer matrices, such as epoxy resin, is critical to optimizing the mechanical, thermal, and electrical properties of the nanocomposites. Due to the tendency of GO to form agglomerates by van der Waals forces, appropriate dispersion strategies are essential to ensure homogeneous distribution and maximize the interfacial interaction between the nanofiller and the matrix.^20–22^

Several dispersion methods have been explored in recent literature. Methods such as high-shear mixing, magnetic stirring, and the use of ultrasonic baths or tips have also been employed, each with specific advantages and limitations.^23–25^ Techniques such as chemical functionalization and the use of solvents or surfactants have been widely explored in an attempt to obtain better dispersion.^26,27^ For example, Yang et al. (2022) investigated the role of chemical functionalization in graphene oxide, highlighting the importance of interfacial interaction for obtaining epoxy nanocomposites with optimized mechanical properties.^28^

Direct dispersion of graphene oxide in epoxy resin, without the use of organic solvents, stands out as a sustainable and efficient approach for the fabrication of advanced nanocomposites. This method avoids the challenges associated with the use of solvents, such as additional costs, environmental risks, and the possibility of nanofiller reassembly during the evaporation process. It also avoids the possibility of residual solvents in the resin, which can interfere with cross-linking, cause defects, and alter mechanical properties, as evidenced by some studies.^29,30^ Furthermore, it promotes a closer interaction between the GO and the polymer matrix, favoring the formation of cross-linked networks that can contribute to improving the final properties of the material.

Studies such as the one carried out by Garg et al. (2018) highlighted the use of fiberglass-reinforced epoxy matrix composites with graphene nanomaterials to improve mechanical properties, such as impact resistance, demonstrating the viability of these materials under demanding conditions of use, with a 21.6% increase in impact resistance.^31^ This wide range of applications reinforces the importance of continuing research aimed at optimizing the dispersion and properties of these materials, to consolidate their position as a key technology for the future.

Despite the extensive research on graphene oxide-reinforced epoxy nanocomposites, a systematic comparison of solvent-free direct dispersion methods under equivalent processing conditions remains scarce in the literature. In particular, the extent to which different dispersion techniques influence graphene oxide distribution and consequently affect the mechanical, thermal, and dynamic-mechanical performance of epoxy nanocomposites has not been fully established. Unlike previous studies that generally investigate a single dispersion route, this work systematically correlates processing methodology with microstructural evolution, interfacial characteristics, and the resulting thermo-mechanical performance of epoxy/GO nanocomposites under identical processing conditions. Therefore, the hypothesis of this study is that dispersion methods capable of promoting better exfoliation and more homogeneous distribution of graphene oxide within the epoxy matrix lead to stronger interfacial interactions and improved overall material performance. To test this hypothesis, five direct dispersion methods—manual stirring, mechanical stirring, magnetic stirring, ultrasonic bath, and ultrasonic tip—were comparatively evaluated using the same graphene oxide concentration (0.5 wt %) and processing conditions. The influence of each technique on dispersion quality and on the resulting mechanical, thermal, and dynamic-mechanical properties of the nanocomposites was systematically investigated.

## 2 Materials and Methods

### 2.1 Materials

LY 1564 epoxy resin, viscosity and density at 25 °C: 1300 mPa.s and 1.15 g.cm^3^, respectively; and XB 3473 hardener, viscosity and density at 25 °C: 100 mPa.s and 1.00 g.cm^3^, respectively from Huntsman were used. Graphene oxide was purchased from NanoView—Applied Nanotechnology. Characteristic chemical composition, determined by elemental analysis, with average contents of carbon between 40–50%, hydrogen between 2–4%, nitrogen between 0–1%, oxygen between 35–45%, and sulfur between 1–3%. Number of layers ranging from 1 to 10.

### 2.2 Resin and Nanocomposite Processing

To manufacture the neat epoxy resin and nanocomposite (NC) specimens, the thermosetting epoxy matrix and hardener were mixed at a ratio of 4:1 w/w, respectively. The mixture was then poured into silicone rubber molds for curing.

For the nanocomposites manufacturing, GO was dispersed in the epoxy resin at a mass fraction of 0.5% using five different methodologies:

1. Manual Dispersion (NC1): Using a mortar and pestle, GO was incorporated into the epoxy resin and dispersed manually until achieving good homogeneity.
2. Mechanical Stirrer Dispersion (NC2): GO was incorporated into the epoxy resin and dispersed using a mechanical stirrer with a propeller at a speed of 1000 rpm for 20 min.
3. Magnetic Stirrer Dispersion (NC3): GO was incorporated into the epoxy resin and dispersed using a magnetic stirrer at a speed of 1000 rpm for 20 min.
4. Ultrasonic Tip Dispersion (NC4): The GO was incorporated into the epoxy resin and dispersed using a Sonics & Materials VC 750 ultrasonic tip at 30% of its maximum operating amplitude for 20 min.
5. Ultrasonic Bath Dispersion (NC5): GO was incorporated into the epoxy resin and dispersed using an UltraCleaner Unique ultrasonic bath operating at 40 kHz and 160 W for 20 min.

During ultrasonic Tip dispersion and ultrasonic bath dispersion, the temperature of the resin was continuously monitored using a thermocouple. To minimize temperature increases caused by ultrasonic tip energy dissipation, the beaker containing the resin was immersed in an ice bath throughout the sonication process, maintaining controlled processing conditions and reducing potential thermal effects on the epoxy system. Under these conditions, the temperature was maintained at approximately (35 ± 5) °C.

Then, for all methodologies used in this work, the hardener was added in the established proportion. After adding the hardener, the nanocomposites were molded by casting in silicone rubber molds and in a vacuum oven at −0.8 bar for 15 min or until the bubbles in the resin disappeared. The materials were cured at 120 °C for 2 h and 180 °C for 4 h.

### 2.3 X-ray Diffractometry

X-ray diffractometry (XRD) was performed at room temperature on a Bruker model V8 Advance Eco, available at UNESP—Guaratinguetá, with a copper (Cu) tube with a wavelength of 1.5418 Å, a voltage of 40 kV, and a current of 25 mA. The scan speed was 10° min^–1^ and the angular increment was 0.01°. The diffractogram was obtained in the region of 2θ = 05–80°. Bragg’s law was applied to calculate the interplanar distance, based on eq 1, and the crystallite dimensions were calculated based on Scherrer’s law expressed in eq 2.

1 nλ=2d⁡sen⁡θ

2 $$D=\frac{K\lambda }{\beta \cos\theta }$$

Where, λ represents the wavelength of the radiation, *n* represents the diffraction order (integer number 1,2,3), *d* refers to the distance between the atomic planes, θ describes the orientation of these planes in relation to the beam, *D* refers to the average diameter of the particles, *K* represents the constant that depends on the shape of the particles, and β is the value of the width at half the height of the diffraction peak.

### 2.4 Fourier Transform Infrared Spectroscopy

Fourier Transform Infrared Spectrometry (FTIR) was performed using a PerkinElmer Spectrum GX FT-IR spectrophotometer and analyzed using the attenuated total reflectance (ATR) method, available at UNESP - Guaratinguetá. The analysis was performed in transmittance mode, over a range of 4000–400 cm^–1^, at a resolution of 4 cm^–1^, and with 64 scans.

### 2.5 Thermogravimetric Analysis

Thermogravimetric analyses (TGA/DTG) were performed on a Seiko Model TG/DTA 6200 from SII Nanotechnology, available at the Department of Materials and Technology (DMT) at UNESP—Guaratinguetá. The following parameters were used for the analyses: mass of approximately 10 mg, platinum sample holder, heating rate of 10 °C min^–1^, oxidizing atmosphere with a flow rate of 100 mL.min^–1^, temperature range from 25 to 1000 °C, and alumina as the reference material. The tests were conducted on GO, on pure epoxy resin without the addition of nanofillers, and on samples with different nanofillers dispersion methods. The objective was to verify the quality of GO, the thermal stability of the resin and composites, and to determine whether the nanofiller dispersion methods would influence the thermal stability of the final composite. Based on eq 3, the heat resistance index *(T*_HRI_ *)* was calculated to evaluate the thermal stability of each nanocomposite and the epoxy resin:^32^

3 $$T_{HRI}=0.49\times \left[T_{5\%}+0.6\times \left(T_{30\%}-T_{5\%}\right)\right]$$

### 2.6 Dynamic Mechanical Analysis (DMA)

The analyses were conducted using a SII Nanotechnology—SEIKO model DMS 6100, available at the Department of Materials and Technology (DMT) at UNESP—Guaratinguetá. The experimental parameters were: three-point bending, nitrogen atmosphere with a flow rate of 100 mL.min^–1^, oscillation amplitude of 10 μm, frequency of 1 Hz, and heating rate of 3 °C.min^–1^. For the test, the samples were manufactured with dimensions of (55 × 10 × 2.5) mm, and a temperature range of 25 to 220 °C.

The reinforcement effectiveness coefficient (*C*) was calculated using eq 4 to evaluate the effect of nanofiller on the storage modulus. Nanofiller is effective when optimal stress transfer occurs at the interface created between the reinforcement and the matrix.

4 $$C=\frac{{\left(\frac{{E^{\prime }}_{g}}{{E^{\prime }}_{r}}\right)}_{Nanocomposite}}{{\left(\frac{{E^{\prime }}_{g}}{{E^{\prime }}_{r}}\right)}_{Epoxy}}$$

Where: *E*′_*g*_ and *E*′_*r*_ are the storage modulus in the glassy and elastomeric regions, respectively. For this study, temperatures of 25 and 195 °C were selected.

The degree of entanglement density (*N*) can be applied to evaluate the dispersion of nanofillers in the polymer matrix. The degree of entanglement density was based on eq 5

5 $$N=\frac{E^{\prime }}{6\mathrm{RT}}$$

Where: *E*′ is the storage modulus at a given temperature, *R* is the gas constant, and *T* is the absolute temperature (K).

The boost efficiency (*r*) is also derived from *E*′ and is calculated according to eq 6

6 $$r=\frac{E_{c}-E_{m}}{E_{m}xV_{f}}$$

Where: *r* is the nanofiller efficiency factor, *E*_*c*_ is the storage modulus of the nanocomposite, *E_m_* is the storage modulus of the epoxy matrix and *V*_*f*_ is the volume fraction of the filler (GO).

The *A* factor was calculated from Tan δ and refers to the existence or not of possible interactions between the polymer matrix and the nanofillers. The A factor is based on the adhesion criteria proposed by Kubat et al.,^33^ where Tan δ can be expressed as a function of the volume fraction and mechanical damping. The A factor was determined using eq 7.

7 $$A=\frac{1}{\left(1-\Phi_{f}\right)}-\frac{{\tan\delta }_{C}}{{\tan\delta }_{m}}-1$$

Where: Φ_*f*_ is the corresponding volume fraction of nanofiller, Tan δ*_C_* corresponds to the nanocomposites, and Tan δ*_m_* to the epoxy matrix.

The dissipation factor (*b*) provides information about the interphase between the polymer matrix and the reinforcement. This parameter assumes that damping depends on the matrix and the interactions and interfaces that tend to form interphase layers trapped between the reinforcement and the matrix. The dissipation factor for nanostructured composites was calculated using eq 8.

8 $$b=\frac{1-\left(\frac{{\tan\delta }_{C}}{{\tan\delta }_{m}}\right)}{V_{f}}$$

Where: *b* is the correction parameter of the reinforcement volume fraction and *V*_*f*_ is the volume fraction of the nanofiller.

### 2.7 Scanning Electron Microscopy (SEM)

The Zeiss EVO LS-15 scanning electron microscope, available at the Materials Imaging Laboratory (LAIMat) of the UNESP Materials and Technology Department, operating in low-voltage mode (SEM-LV), was used to analyze the morphological aspects of graphene oxide and nanocomposites. For the nanocomposites, gold deposition of approximately 30 nm was performed by plasma sputtering on the samples using the Quorum Technology equipment, model Q150RES.

### 2.8 Transmission Electron Microscopy (TEM)

To verify the dispersion of graphene oxide in the matrix, the nanocomposites were characterized by transmission electron microscopy using a 120 kV Tecnai Spirit transmission electron microscope (TEM). The samples were first sectioned with ultrathin sections using a Leica EM UC7 ultramicrotome. The images were obtained at the Institute of Advanced Marine Studies of the São Paulo State University (UNESP) in São José dos Campos, São Paulo.

### 2.9 Impact Resistance

The impact resistance test was performed using the Izod methodology based on ASTM D256 standard and a 0.5 J hammer, available in the Materials and Technology Department at UNESP in Guaratinguetá. Five samples of neat epoxy resin and nanocomposites were tested.

## 3 Results and Discussion

### 3.1 Characterization of Graphene Oxide

Figure 1 shows the X-ray diffractogram obtained from GO, with each peak corresponding to a crystallographic plane. An intense peak at 2θ near 10° was observed; this peak corresponds to the (002) plane of graphite. This result indicates that the oxidation process was successful, since in graphite (the precursor material), this plane is located near 26°. Through this analysis, using Bragg’s law, the interplanar distance of GO was approximately 0.8845 nm, significantly higher than that of graphite, which, according to the literature, has an interplanar distance close to 0.342 nm.^34^ This increase confirms the efficiency of the oxidation process, since the interplanar distance of the nanosheets increased due to the incorporation of oxygenated groups during the oxidation process.^35^ The XRD technique also allowed estimation of the average crystallite size of GO. Using Scherrer’s equation, the material was found to have an average diameter of 7.8921 nm, corresponding to approximately nine stacked layers.

**Figure 1:**
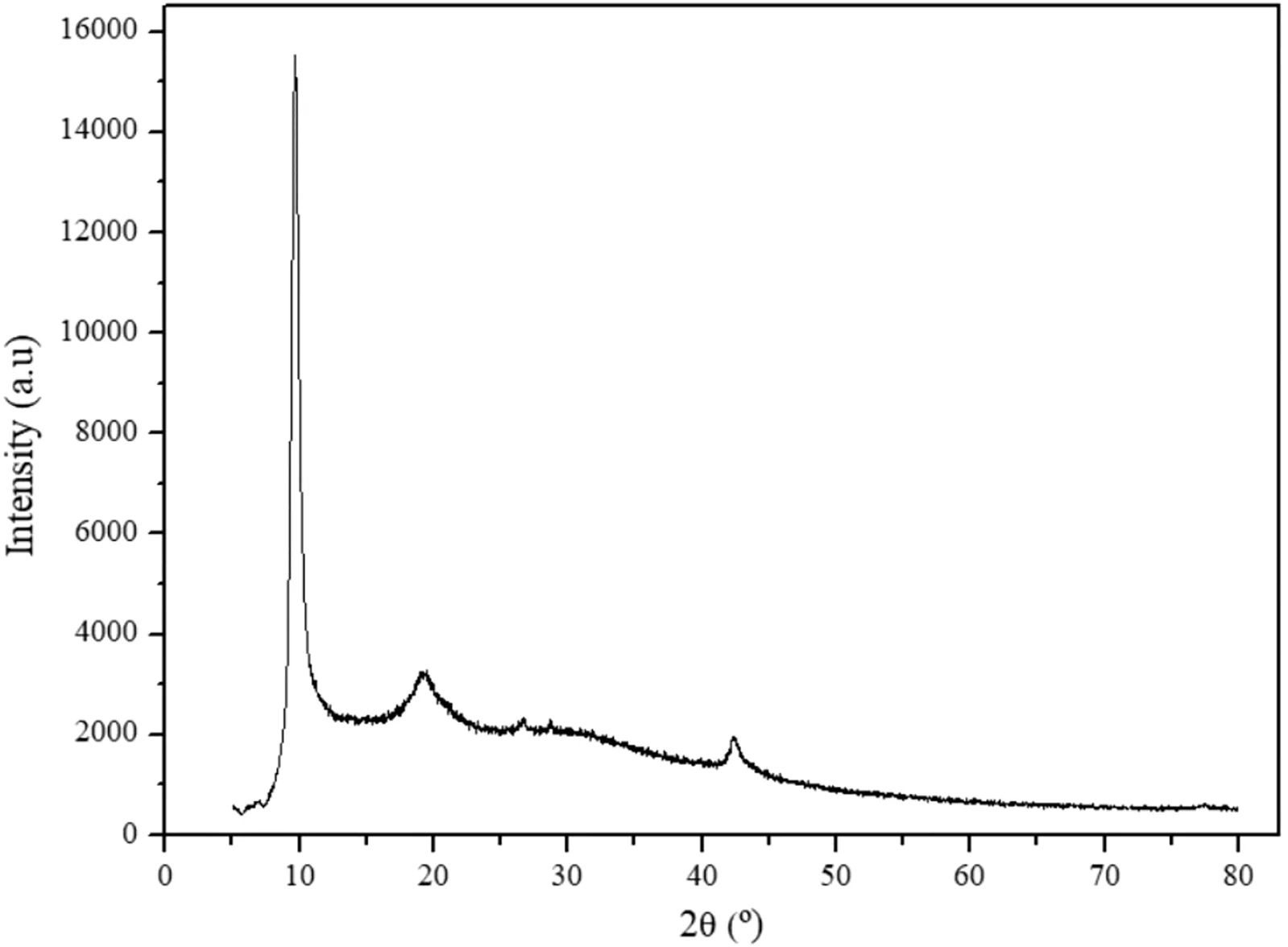
XRD of graphene oxide.

Figure 2 shows the Fourier Transform Infrared Spectroscopy of GO. This characterization is important for assessing the success of the oxidation and functionalization processes, as evidenced by the presence of oxygenated groups in the GO structure, including hydroxyls, carbonyls, epoxy, and carboxyl.

**Figure 2:**
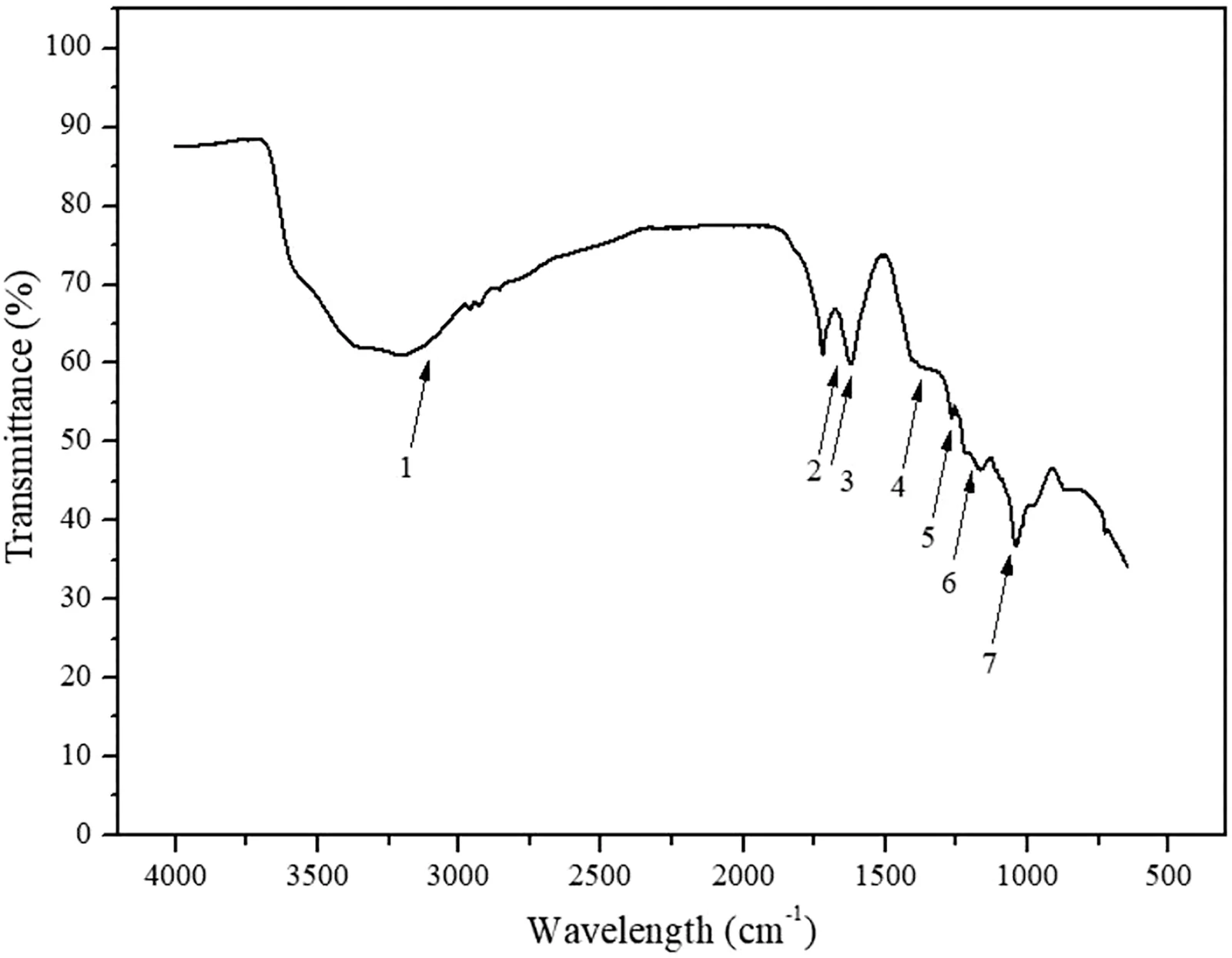
FTIR of graphene oxide.

The results confirm the effectiveness of the GO synthesis process, as demonstrated by the presence of characteristic bands associated with functional groups formed during the graphite oxidation process. Band (1), located at ∼3440 cm^–1^, corresponds to the stretching vibration of the hydroxyl group (O–H). Band (2), at ∼1720 cm^–1^, refers to the stretching vibrations of C = O. Band (3), at 1627 cm^–1^, is characteristic of C=C stretching. Band (4), at 1370 cm^–1^, corresponds to the C–O stretching vibration of epoxy groups. Band (5), at 1230 cm^–1^, indicates the stretching vibration of the C–O bonds from carboxylic acid. In the region between 1300 and 1000 cm^–1^, bands (6) and (7) appear, which are associate with the C-Ostretching vibrations of ester groups.^31,36,37^

The curves depicted in Figure 3 were from the thermogravimetric analysis (TGA) of GO, illustrating the variation in mass as a function of temperature. GO exhibits at least four distinct stages of thermal decomposition and a low residual mass, indicative of a high degree of purity. The temperature corresponding to the maximum degradation rates, defined from the peaks of the DTG curve, were approximately 60 °C, 185 °C, 270 °C, and 500 °C. This analysis provides insight into the thermal stability of the material and serves as an important parameter for its characterization.

**Figure 3:**
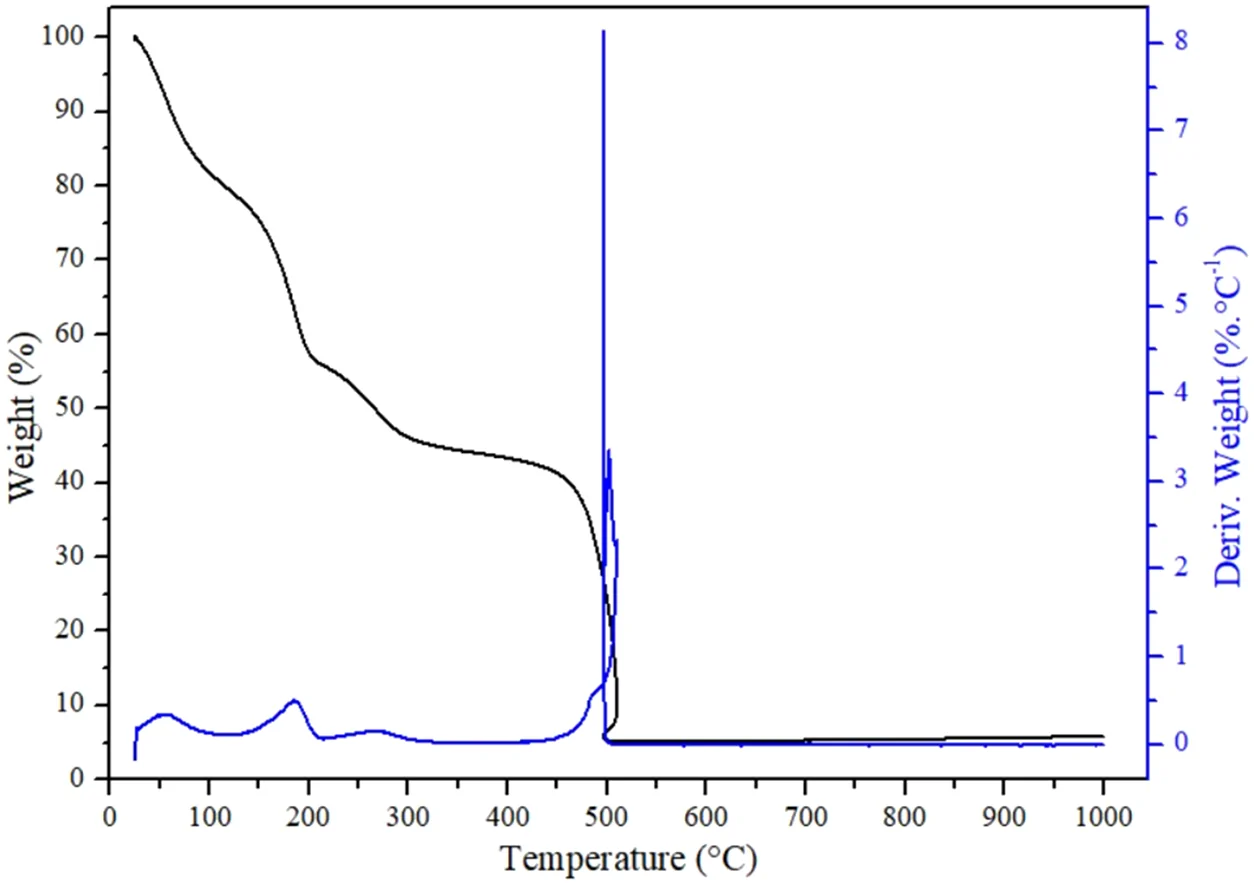
Thermogravimetric analysis of graphene oxide.

The main mass loss events of GO can be correlated with its thermal degradation profile. The initial mass loss of approximately 15% below 100 °C refers to the removal of adsorbed water. The subsequent 40% mass loss between 100 and 320 °C occurs due to the decomposition of oxygen-containing functional groups. Finally, the mass loss of 45% from 320 to 1000 °C is related to the oxidative pyrolysis of the carbon structure.^39,40^

The carbon combustion temperature is prominently indicated by the peak in the DTG curve at around 500 °C. At this stage, the system releases significant energy, causing a localized heat effect that leads to a temperature rollback in the recorded curve, reflecting the difference between furnace and sample temperatures. Compared to other graphitic nanofillers, such as graphene, GO exhibits a lower thermal degradation threshold due to its high density of oxygenated groups.^39^ Additionally, GO requires less thermal energy to overcome the weaker, nongraphitic sp^3^-hybridized carbon bonds (a characteristic hybridization of GO due to the functionalization of graphene layers), which contains a high density of defects resulting from the oxidation reaction. In contrast, graphene requires higher temperatures because they demand a greater amount of thermal energy to break the sp^2^-hybridized carbon atoms, which are ordered through covalent bonds in a hexagonal carbon structure.^41^

Figure 4 presents the scanning electron microscopy (SEM) micrographs for graphene oxide nanosheets. The analysis revealed typical features of lamellar and wrinkled structures, consistent with the literature for graphene-derived materials, which exhibit thin sheets with partial overlapping and distinctive of characteristic wrinkles and folds.^38^ These deformations are attributed to the high surface-to-volume ratio of GO, and to internal stresses generated during drying and/or synthesis processes.

**Figure 4:**
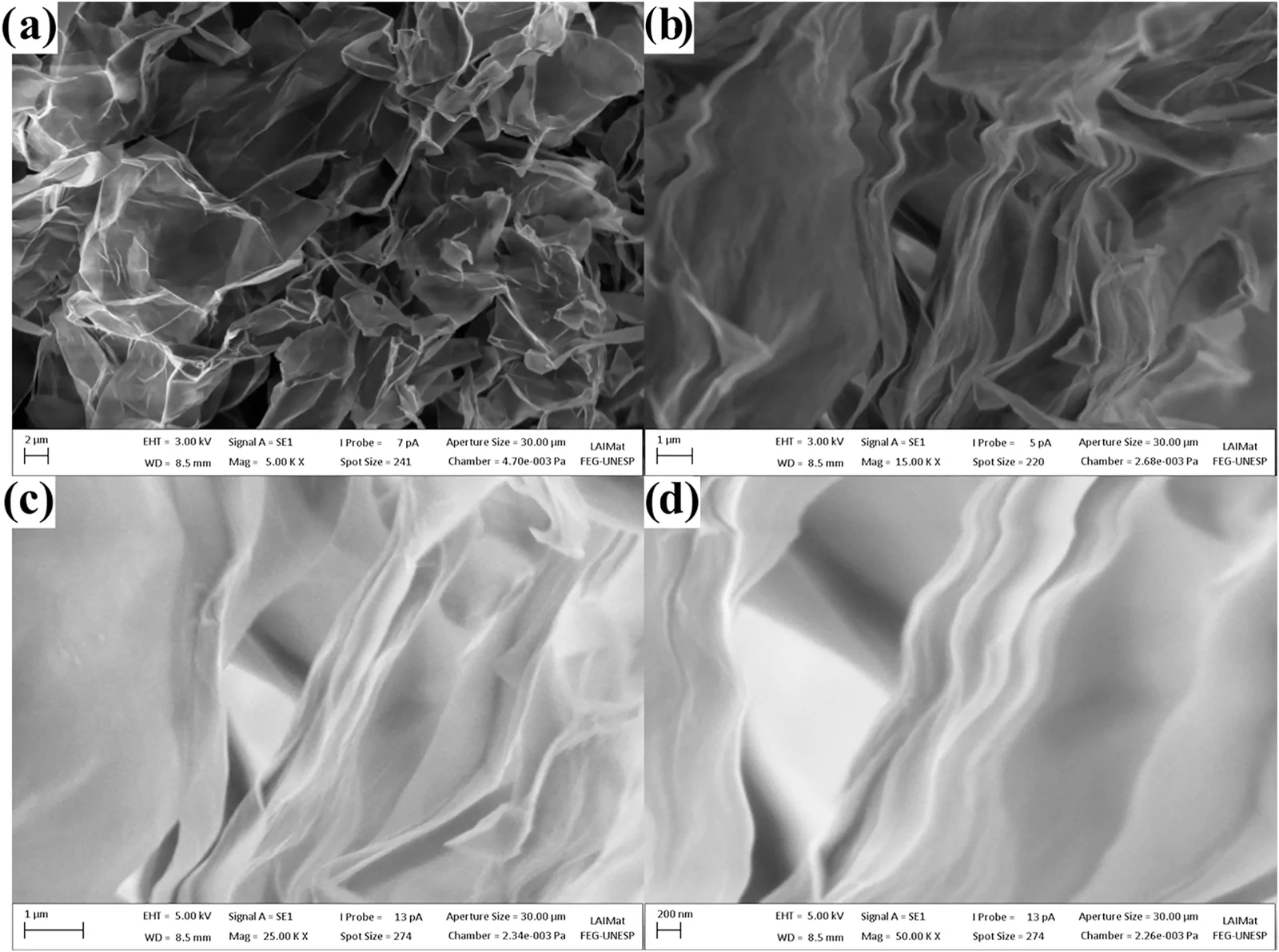
Scanning electron microscopy (SEM) micrographs of graphene oxide nanosheets at different magnifications: (a) 5.000×, (b) 15.000×, (c) 25.000×, and (d) 50.000×.

At 5.000× magnification (Figure 4a), a three-dimensional network of interconnected sheets is observed, showing partial stacking and a relatively homogeneous nanosheet distribution. This three-dimensional architecture is desirable for applications requiring high surface area. At higher magnifications of 15.000× and 25.000× (Figure 4b and c), the ripples and folds of the sheets become more pronounced attributed to the presence of oxygen-containing functional groups introduced during the graphite oxidation process. These wrinkles enhance the surface area and facilitate interfacial interactions with other phases, such as polymer matrices in composites. At 50.000× magnification (Figure 4d), the surface texture of the sheets becomes clearer, revealing nanometric ripples. The smooth yet undulated surface indicates that the GO sheets are thin and well-exfoliated. The absence of dense agglomerates at this scale suggests good material dispersion, which is essential for preserving the individual properties of the sheets in hybrid systems.

### 3.2 X-ray Diffractometry of Epoxy and Nanocomposites

The XRD patterns of the nanocomposites shown in Figure 5 indicate the absence of a distinct diffraction peak associated with graphene oxide within the epoxy matrix. However, this observation should be interpreted with caution, since the absence of a detectable GO reflection may result from multiple factors, including exfoliation and homogeneous dispersion of GO sheets and the relatively low GO concentration employed. This result may indicate that the nanofiller is well dispersed in few layers and small sizes, preventing the formation of well-defined diffraction peaks, as found in other study.^42^ The results also show that the dispersion methods influenced the XRD intensity when compared with neat epoxy resin. Upon incorporation of GO, a reduction in the intensity of the resin halo was observed, especially for NC4. The presence of GO in the matrix may be absorbing or blocking some of the X-ray radiation, explaining the reduction in the intensity of the epoxy resin peak. The lower intensity observed for NC4 may be associated with a more homogeneous distribution of the nanofiller throughout the matrix; however, XRD alone is insufficient to conclusively confirm this effect. Therefore, the assessment of dispersion quality was supported by complementary SEM and TEM analyses, as well as by the mechanical and dynamic-mechanical results. Overall, the result indicates that GO is uniformly whiting the epoxy, without creating new crystalline phases or aggregates large enough to produce well-defined reflection or diffraction peaks.

**Figure 5:**
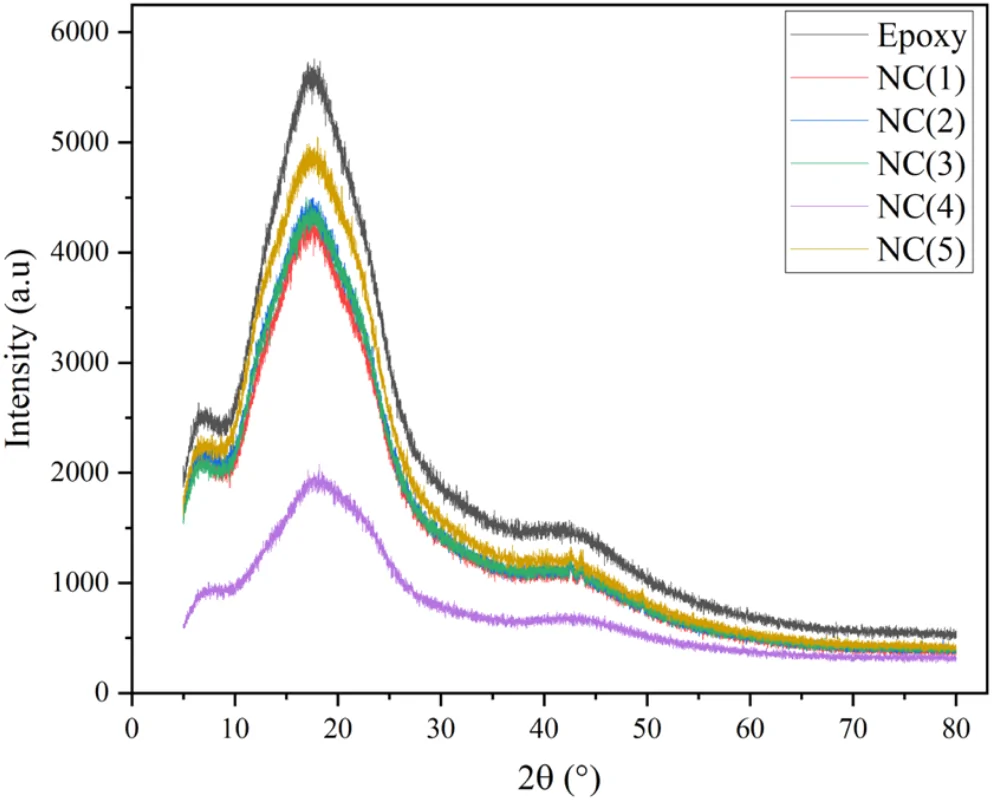
X-ray diffractometry of pure epoxy resin and processed nanocomposites.

### 3.3 Fourier Transform Infrared Spectroscopy of Epoxy and Nanocomposites

Figure 6 shows the FTIR spectrum of the pure resin in comparison with the nanocomposites The spectra of nanocomposites exhibit a band at ∼1733 cm^–1^, corresponding to the C=O stretching of carboxylic groups of GO.^25^ An increase in intensity whitin 3200–3600 cm^–1^ range suggests the formation of hydrogen bonds between GO and hydroxyl groups of the resin.^29^ The cured epoxy resin shows characteristic absorption bands at 3397 cm^–1^ (O–H stretching from hydroxyl groups) and 3050 cm^–1^ (C–H stretching of the epoxy). Bands at 1607, 1580, and 1508 cm^–1^ are attributed to the aromatic rings of the epoxy structure.^43^ The bands at 1248 cm^–1^ and 935 cm^–1^ correspond to the symmetric stretching of the epoxy ring and the C–O stretching of the epoxy group, respectively. The peak at 827 cm^–1^ is assigned to out-of-plane bending of aromatic C–H (phenyl).^31^ Overall, no major differences were observed between the pure epoxy resin and the nanocomposites in the other absorption bands, likely due to the low GO content (0.5 wt %) in the epoxy matrix.

**Figure 6:**
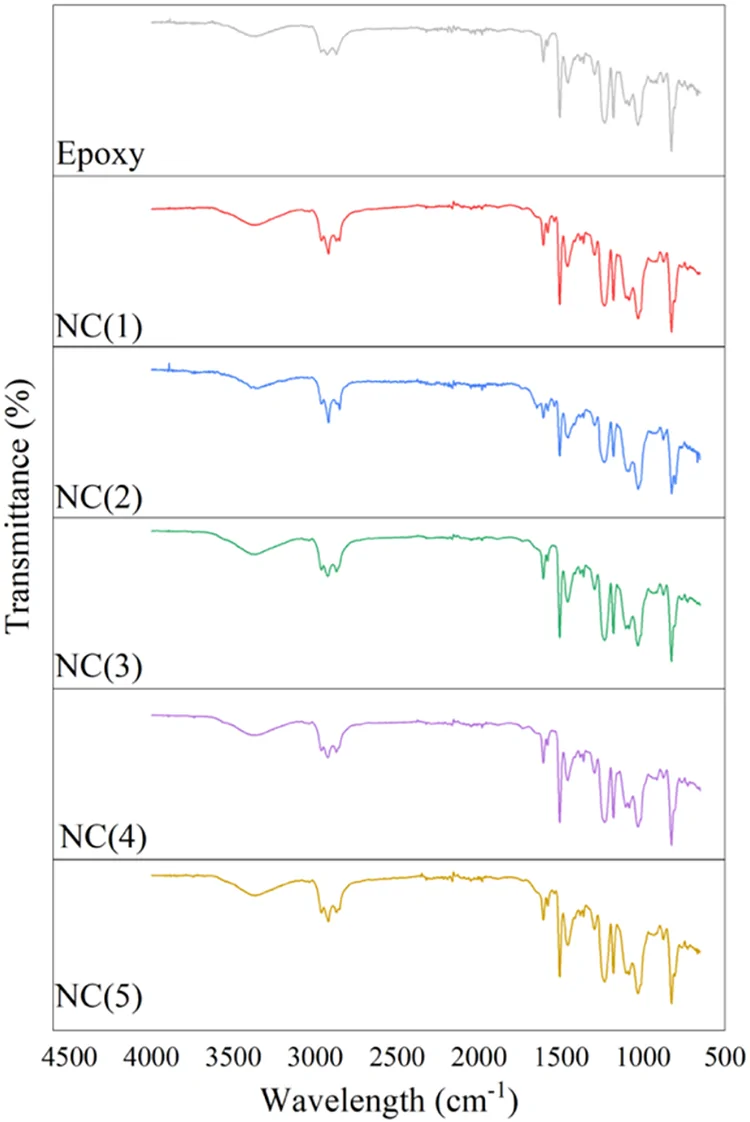
FTIR of the pure epoxy resin and the manufactured nanocomposites.

### 3.4 Thermogravimetric Analysis of Epoxy and Nanocomposites

Figure 7 presents the thermogravimetric results of the pure epoxy resin and the epoxy/GO nanocomposites manufactured using different dispersion methods. Figure 7(a) shows the mass variation as a function of temperature. Both the neat resin and the nanocomposites exhibit three stages of thermal decomposition. The temperature of maximum degradation rate, defined by the peak of the DTG-derived thermogravimetric curves (Figure 7(b)), was not significantly affected by the incorporation of GO or the dispersion methods used; its value was approximately 385 °C, due to the degradation of the epoxy network.^44^ This analysis revealed the thermal stability temperature of the resin and nanocomposites. Therefore, applications should be limited to temperatures below the material’s onset of degradation, around to 285 °C. The results are consistent with values reported in other studies employing epoxy resins as a matrix for structural composites.^45^

**Figure 7:**
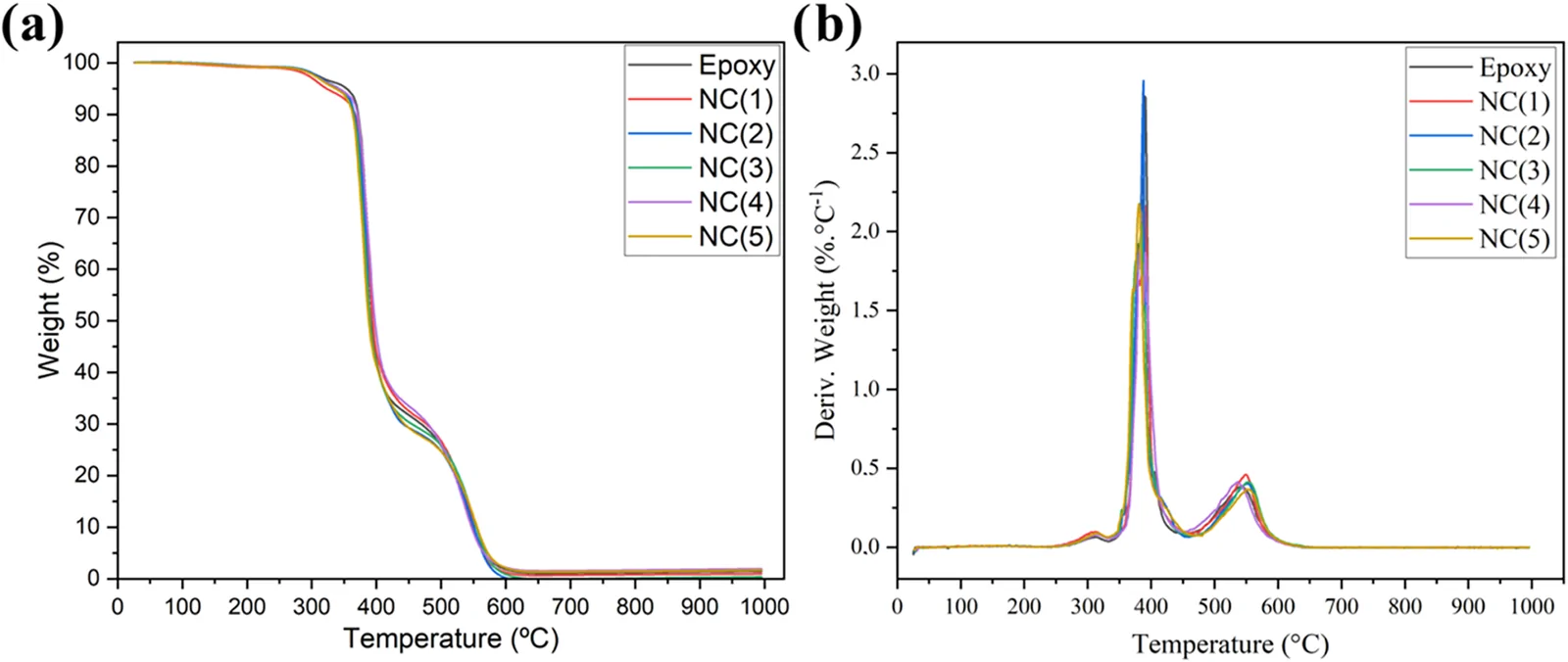
Thermogravimetric analysis of epoxy/GO nanocomposites (a) TGA and (b) DTG.

As shown in Table 1, the initial degradation temperature (*T*_5%_) was subtly influenced by the dispersion methods used. The nanocomposites showed a slightly lower initial thermal stability than the pure resin, which may be associated with the presence of oxygenated groups present in greater quantities in these materials. On the other hand, sample NC4 showed a slight increase in temperature corresponding to 30% mass loss (*T*_30%_), a behavior not observed for other dispersion methods. These results suggest that the addition of GO induces a tortuous path effect within the matrix, whereby better nanosheet dispersion enhances the material’s thermal stability. This improvement may occur due to the delay in the escape of degradation products, reduced volatile diffusion, hindered oxygen permeation, and increased char formation, respectively.

**Table 1:** Data Obtained through TGA for Pure Epoxy and Nanocomposites

Sample	Residue at 950 °C (%)	*T*_5%_ (°C)	*T*_30%_ (°C)	*T*_HRI_
Epoxy	1.49	350.28	393.13	184.23
NC1	0.97	322.87	394.14	179.16
NC2	0.12	345.31	390.07	182.36
NC3	0.37	337.39	390.08	180.81
NC4	1.97	345.32	400.1	185.31
NC5	1.67	336.48	387.56	179.89

The heat resistance index (*T*_HRI_) has emerged as an accurate parameter for assessing the impact of nanoparticles on the thermal stability of polymer composites. This approach is based on the correlation between specific temperatures at defined points of thermal degradation, which allows determining whether the nanocomposite presents greater stability compared to the pure polymer. Since (*T*_HRI_) is directly proportional to thermal stability, higher values indicate superior resistance to thermal degradation. In general, incorporating inorganic nanoparticles into polymer matrices enhances the thermal stability of the nanomaterials. However, this effect strongly depends on factors such as good dispersion, filler/matrix interactions, and barrier effects.^46^ In the present study, only subtle differences were observed along the samples.

### 3.5 Dynamic Mechanical Analysis of the Nanocomposites

The curves obtained from the results of the dynamic mechanical analysis for epoxy resin and for the nanocomposites, with different dispersion methods, are shown in Figure 8. A characteristic temperature-dependent behavior was observed: the storage modulus (*E*′) (Figure 8(a)), decreased with increasing temperature, while both the loss modulus (*E*″) and the damping factor (tan δ or tan delta) initially remained nearly constant and then increase as the temperature rose (Figure 8(a,b)).

**Figure 8:**
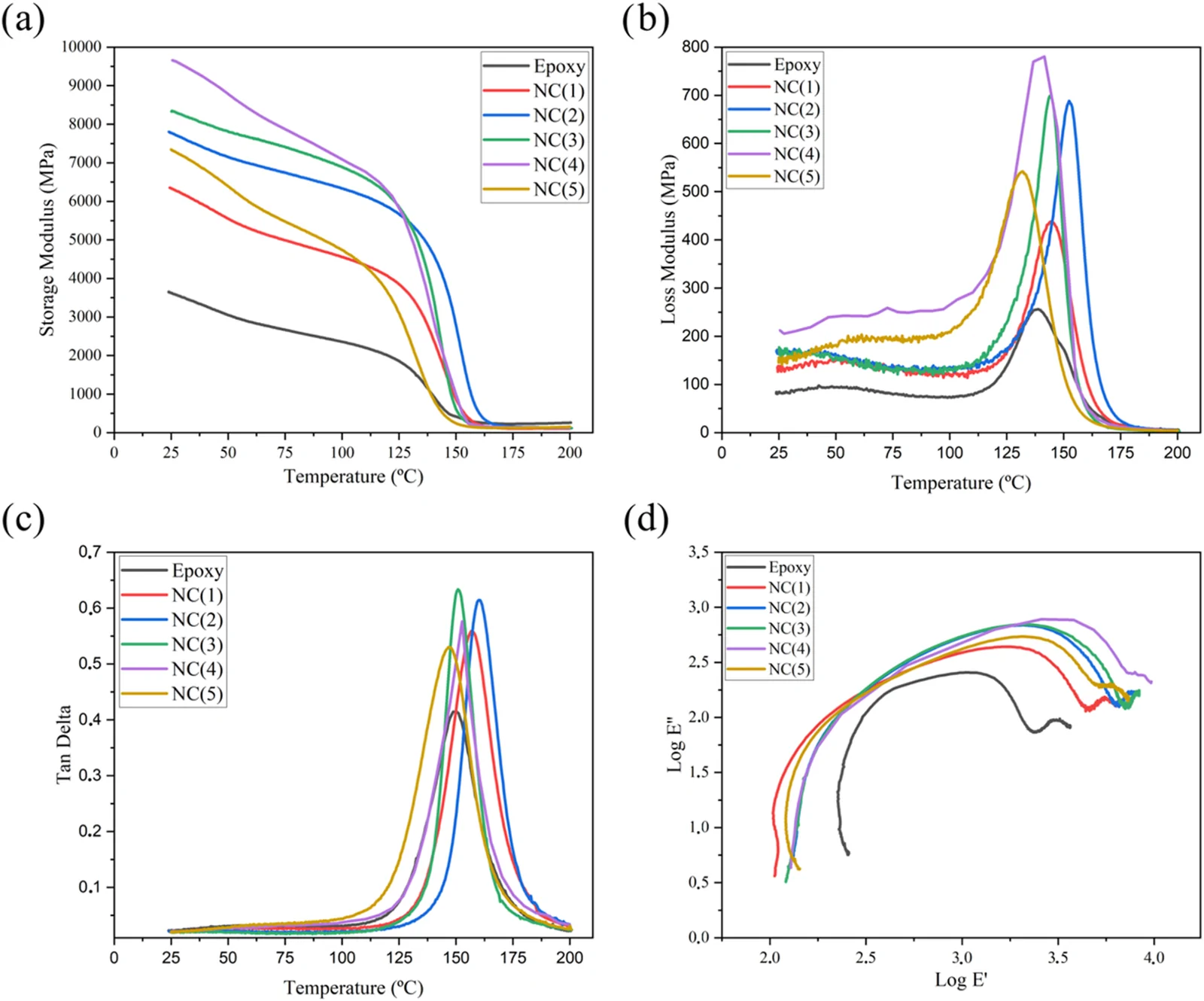
DMA curves: (a) storage modulus, (b) loss modulus, (c) tan δ, and (d) Cole–Cole for the epoxy/GO nanocomposites.

According to Figure 8(a), both the addition of GO and the dispersion method significantly influenced the storage modulus values. Near room temperature (25 °C), Whitin the glassy region, the incorporation of GO into the epoxy resin led to the highest storage modulus values, indicating a stiffening effect caused by the incorporation of nanofillers. Among the different dispersion methods, manual dispersion (NC1) resulted in the lowest storage modulus (6315 MPa), followed by ultrasonic bath (NC5—7026 MPa), mechanical stirring (NC2—7767 MPA), magnetic stirring (NC3—8319 MPa), and ultrasonic-tip dispersion (NC4—9660MPA).

The *E*′ is a key parameter for material applications, as it represents the elastic response under applied load. The observed difference in the *E*′ values is probably due to the difference in the dispersion method used. Properly dispersed nanofillers tend to improve interfacial interactions within the matrix, enhancing load transfer and stiffness, which justifies the superior modulus of the NC4 nanocomposite. The remarkable 157% increase in storage modulus observed for NC4 can be explained by molecular-level interactions occurring at the graphene oxide/epoxy interface. The improved exfoliation and homogeneous distribution of graphene oxide increase the available interfacial area, allowing a larger fraction of oxygen-containing functional groups to interact with the epoxy network. These intermolecular interactions, particularly hydrogen bonding and dipolar interactions, restrict the mobility of polymer chains within the interphase region, thereby increasing stiffness. Simultaneously, the enlarged interfacial area promotes more efficient stress transfer from the epoxy matrix to the graphene oxide nanosheets, maximizing the reinforcing effect of the nanofiller. Therefore, the observed improvement is attributed not to the intrinsic stiffness of graphene oxide alone, but to the synergistic effect of enhanced dispersion, stronger interfacial interactions, and effective load transfer throughout the nanocomposite.

It can be observed that the NC4 sample shows a more pronounced reduction in modulus compared to the other nanocomposites. Samples NC1, NC2, NC3, and NC5 showed a more stable drop in this temperature range compared to the NC4 sample, which may be related to a higher content of agglomerated GO nanoparticles, possibly indicating that they acted as a barrier, restricting the movement of the polymer chains and reducing the slope of the curve in this region.

The NC1 sample showed a lower capacity to store energy compared to the other nanocomposites, possibly due to poorer homogeneity of the nanofiller dispersion in the polymer matrix, as it was manually dispersed. However, the dissipation of the stored energy occurred more gradually over time and temperature. In the elastomeric region, the *E*′ of all samples did not vary significantly remaining close to 125 MPa, indicating that the dispersion method had little influence in this region.

In Figure 8(b) displays the loss modulus of the samples. The behavior exhibited by the materials shows that the dispersion method and the addition of GO influenced the results. In the glassy region, the nanocomposites showed higher modulus values than the neat resin. The peak of the *E*″ corresponds to the maximum energy dissipation, which is associated with polymer chain mobility in the glass transition region. NC4 sample showed the highest modulus in this region, supporting the earlier discussion regarding the storage modulus, the greater energy storage capacity shown by the NC4 sample, as well as the more abrupt reduction in modulus, led to greater energy dissipation. Therefore, the loss modulus in this region was higher.

Figure 8(c) presents the behavior of the samples in terms of loss tangent. All samples exhibited a characteristic peak in the glass transition region. Analysis of this peak allowed determination of the glass transition temperature of the composites and maximum tan δ values, which are summarized in in Table 2.

**Table 2:** Data Obtained from the Dynamic Mechanical Analysis of the Fabricated Nanocomposites: Factor (*C*), Reinforcement Efficiency (*r*), Degree of Entanglement (*N*), Factor (*A*), Factor (*b*)

Sample	*E*′_g 25 °C_ (MPa)	*E*′_r 195 °C_ (MPa)	Peak height (tan δ)	*T*_g_ (°C) Tan δ	*C*	*N*	*r*	*A*	*b*
Epoxy	3760.80	252.01	0.4142	149.57	-	-	-	-	-
NC1	6315.79	94.39	0.5579	157.04	4.48	0.42	1.38	0.8532	0.1467
NC2	7767.4	118.56	0.6286	160.77	4.39	0.52	2.13	0.8506	0.1499
NC3	8319.19	129.34	0.6335	151.16	4.31	0.56	2.42	0.8471	0.1528
NC4	9660.45	151.03	0.5852	152.80	4.28	0.65	3.14	0.8315	0.1684
NC5	7026.03	105.51	0.5262	147.42	4.46	0.47	1.73	0.8507	0.1493

The observed difference, even if small, may be due to the different dispersion methods used during the fabrication step, which in turn affect the homogeneity of the system. This difference is related to the ability of each sample to store and dissipate energy. The samples that exhibited smoother energy dissipation in the glassy region showed a more pronounced shift in the transition temperature range compared to the others. The nanofiller dispersed using the ultrasonic tip, (sample NC4) enabled greater amount of energy accumulation; however, the nanofiller dispersed by this method was unable to retain that energy, resulting in faster dissipation.

It is important to highlight that the relaxation process in polymers and composites is highly complex and depends on several factors, including applied stress, particle/matrix interaction, temperature, etc. Several studies have found that the reinforcement effects observed in the glassy region do not necessarily correspond to a shift toward higher glass transition temperatures, consistent with the findings of this study.^42^

Figure 8d shows the Cole–Cole plot, which provides insight into the distribution of molecular relaxation times and material homogeneity. As expected, all curves exhibited a semicircular shape. The incorporation of GO, as well as the dispersion methods employed, altered the material’s behavior. Closer semicircles indicate that the relaxation process occurs over a narrower range. Compared to neat epoxy, the nanocomposites exhibited a broader relaxation process, which may be attributed to changes in the segmental mobility of the polymer matrix caused by interactions between the nanofiller and the epoxy chains.^47^

The calculated effectiveness coefficient (*C*) is presented in Table 2. The *C* values of the nanofillers in the samples were similar; the lowest values were obtained for the nanocomposites produced by methods NC4, NC3, NC2, NC5, and NC1 These results indicate that the dispersion methods were effective. In particular, the lowest C value observed by sample NC4 suggests good dispersion of GO whitin the polymer matrix, reflecting nanofiller effectiveness.

Table 2 also presents the entanglement density degree (*N*) of each nanocomposite. This parameter was used to evaluate the dispersion of GO within the polymer matrix. Higher *N* values suggest lower interaction between the nanofillers,^48^ which reduces the tendency for agglomeration. The nanocomposite produced by the NC4 method showed the highest *N* values, followed by NC3, NC2, NC5, and NC1, respectively. This trend suggests a higher degree of dispersion for NC4, which may be related to the efficiency of its dispersion method. Improved dispersion corresponds to weaker interlayer interactions among the GO sheets.

A similar behavior was observed for the reinforcement efficiency factor (*r*), where NC4 had the highest value, followed by NC3, NC2, NC5, and NC1, respectively. Consistent with the entanglement density results, this suggests that the NC4 method is the most effective for dispersing GO.

The adhesion factor (*A*), obtained from the tan δ values at 25 °C, is also reported in Table. As noted in other studies,^49^ low adhesion factor values indicate a high degree of interaction or adhesion between the matrix and the nanofiller. In the present study, nanocomposite NC4 presented the lowest A value, suggesting improved interfacial adhesion between the epoxy matrix and GO. Conversely, NC1 showed the highest adhesion factor, which may be related to the manual dispersion method used in its preparation, which led to poorer phase interaction. The sequence of adhesion factor values—NC1, NC5, NC2, NC3, and NC4—further supports this interpretation.

The dissipation factor (b) is also reported in Table 2. For values of *b* < 0, the interface is considered weak, whereas *b* > 0 corresponds a strong interface.^50^ According to this criterion, all nanocomposites presented strong interfaces, as all values of the b parameter were above 0. Among them, NC4 shower the highest b value, indicating the formation of a more solid and robust interfacial region. These results suggest that nanocomposite NC4 possesses a greater number of effective interfacial regions, which may enhance micromechanical stress transfer within the composite.

Figure 9 represents the influence of temperature on the factors calculated from dynamic mechanical analysis data. In general, the storage modulus (Figure 9a) for all materials decreased with increasing temperature, a typical behavior of polymeric materials due to the enhanced chain mobility. Despite this reduction, the nanocomposites maintained higher modulus than the pure resin, indicating that GO continued act as reinforcement by partially restricting chain movement. In the difference in *E*′, however, did not remain constant as the temperature increased. Around 100 °C, NC4 exhibited values similar to NC3, suggesting that the dispersion method had little influence on modulus at higher temperatures. The efficiency between these two methods was very close (±195%). Although the reinforcement effect was still present, it was attenuated due to the increased segmental mobility of the matrix.

**Figure 9:**
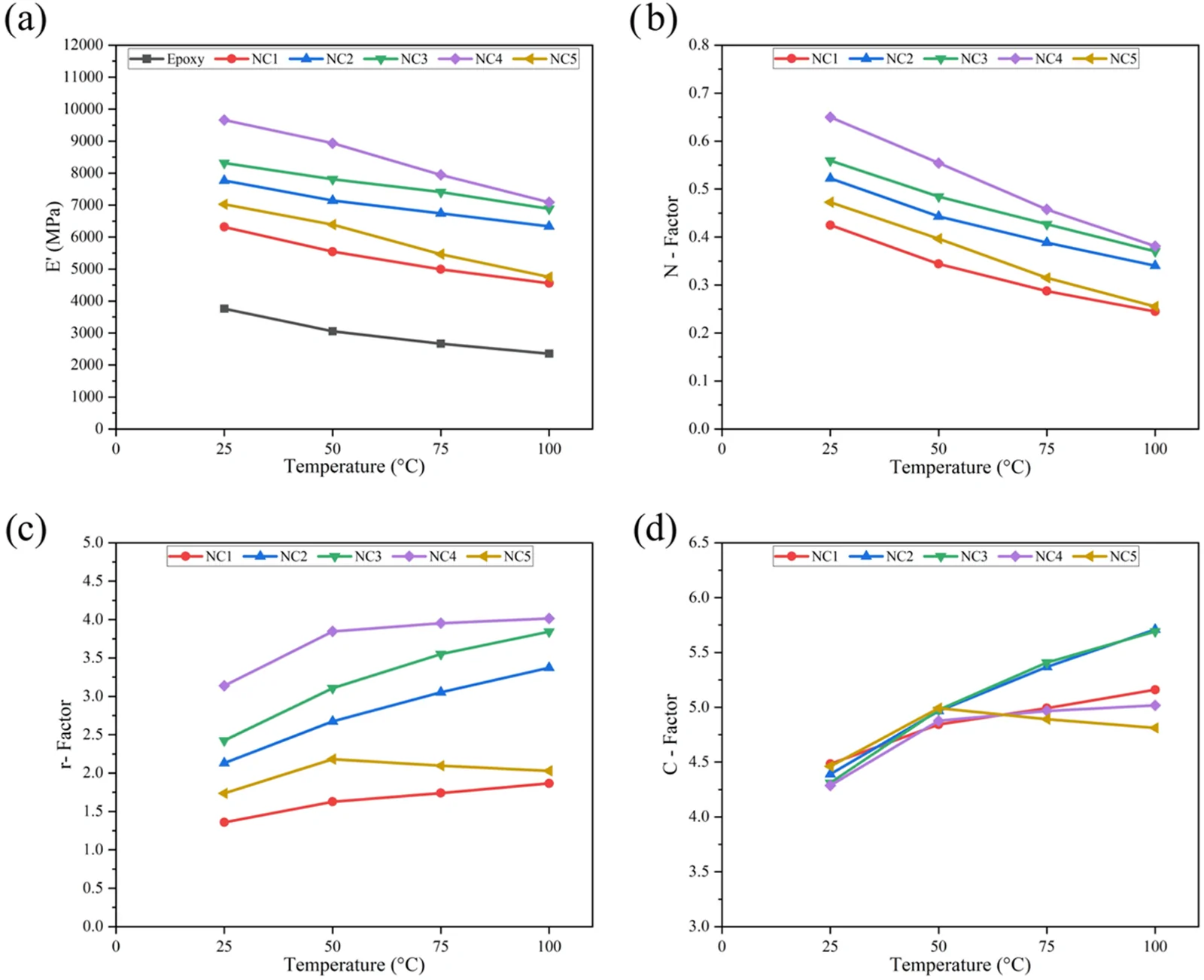
Influence of temperature on the factors obtained from dynamic mechanical analysis: (a) Storage modulus (*E*′), (b) entanglement density degree (*N*), (c) reinforcement efficiency factor (*r*), and (d) effectiveness coefficient (*C*).

The entanglement density degree (*N*) also decreased with increasing temperature (Figure 9b), reflecting stronger nanofiller-nanofiller interactions as chain mobility increased. Regarding the different dispersion methods, at temperatures close to room temperature, a more pronounced difference was observed, with NC4 presenting the highest value. At higher temperatures, around 100 °C, NC4 showed a more significant decrease in the *N* factor, approaching the values of NC2 and NC3. These results indicates that dispersion methods have greater influence at lower temperatures, whereas at higher temperatures the mobility of GO whitin the softened matrix allows greater interaction among nanofillers.

The result of the reinforcement efficiency factor (*r*) as a function of temperature is shown in Figure 9c. This analysis allowed verifying the importance of incorporating GO into the epoxy matrix. In general, the dispersion method used influenced the results. At temperatures near room temperature, the difference was more significant, with NC4 presenting the highest value. With increasing temperature, an improvement in efficiency was observed for all composites due to the incorporation of GO, since the calculation of the r factor considers the value presented by the pure resin. This analysis was important to understand that although the method used to produce NC4 showed superior values to the others at room temperature, its value remained more constant with increasing temperature. For the other methods, especially NC2 and NC3, a more significant increase was observed, approaching the value presented by NC4 around 100 °C.

The effectiveness coefficients (*C*) calculated at different temperatures are presented in Figure 9d. In general, *C* values increased with rising temperature, and their values increased due to the lower efficiency of GO within the matrix.

### 3.6 Transmission Electron Microscopy (TEM)

The results allowed visualization of microstructural in GO and the nanocomposites according to the different dispersion methods. Figure 10a shows the TEM image of the neat epoxy matrix, without the GO. Figures 10b–f present the images of the nanocomposites, highlighting the presence of GO in the epoxy matrix, and how the dispersion methods influenced the morphology of the nanocomposites. In general, GO appeared as a collection of multiple graphene sheets stacked together. Its multilayer structure was evident from regions of lighter contrasts. Figure 10b and 10c revealed a considerably higher number of overlapping GO sheets compared to the other samples.

**Figure 10:**
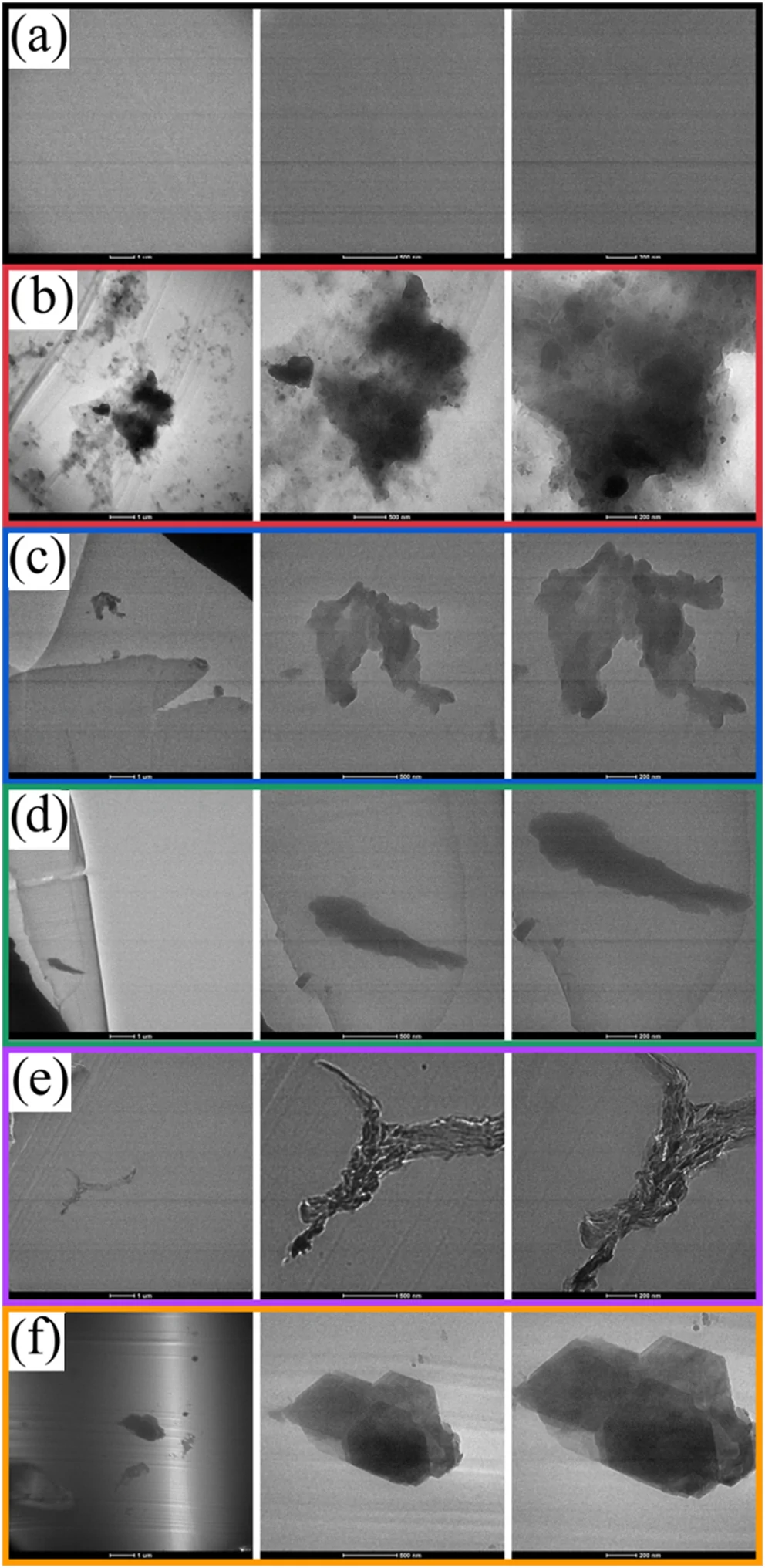
Transmission electron microscopy (TEM) images showing the morphology and dispersion of GO sheets in the epoxy matrix according to the different dispersion methods: (a) neat epoxy, (b) NC1, (c) NC2, (d) NC3, (e) NC4, and (f) NC5.

The NC4 sample (Figure 10e) displayed different levels of transparent sheet-like arrangements characteristic of GO (Figure 4), confirming that the thickness of GO layers depends on the dispersion method employed. This was inferred from the transparency of the exfoliated GO nanosheets compared to the opacity of the image, since the electron beam cannot penetrate thick sections. The different shades, from transparent to black (denser regions), for GO suggest that one or multiple GO sheets are stacked on top of each other, respectively. GO was observed to be randomly dispersed throughout the cured matrix, dispersion methods influencing both exfoliation and spatial distribution of nanosheets. GO is visible as flat sheets when positioned longitudinally to the ultrathin cut surface and as fine lines when transversal to the surface. This feature was also observed in other studies.^43^

The direct incorporation method proved to be efficient, as the dispersion of nanomaterials in solvents typically causes the GO sheets to restack after drying, forming multilayers. Dispersion using an ultrasonic tip contributed to more effective exfoliation, as indicated by its lighter color compared to the other samples. In contrast, manual dispersion exhibited darker regions, suggesting areas with stacked GO sheets. Mechanical and magnetic also demonstrated efficiency in promoting GO exfoliation. Regarding the homogeneity of the nanosheets whiten the matrix, the results indicate that the nanofiller is more uniformly distributed in the epoxy matrix of NC4, as shown in Figure 10e.

### 3.7 Impact and Fractographic Analysis by SEM

The mechanical behavior of epoxy/GO nanocomposites under impact was evaluated based on the results presented in Figure 11 and complemented by the fractographic analysis of fracture surfaces using scanning electron microscopy (SEM), shown in Figure 12. The correlation between the mechanical data and the morphology of the fractured surfaces made it possible to understand the energy dissipation mechanisms associated with each graphene oxide dispersion method.

**Figure 11:**
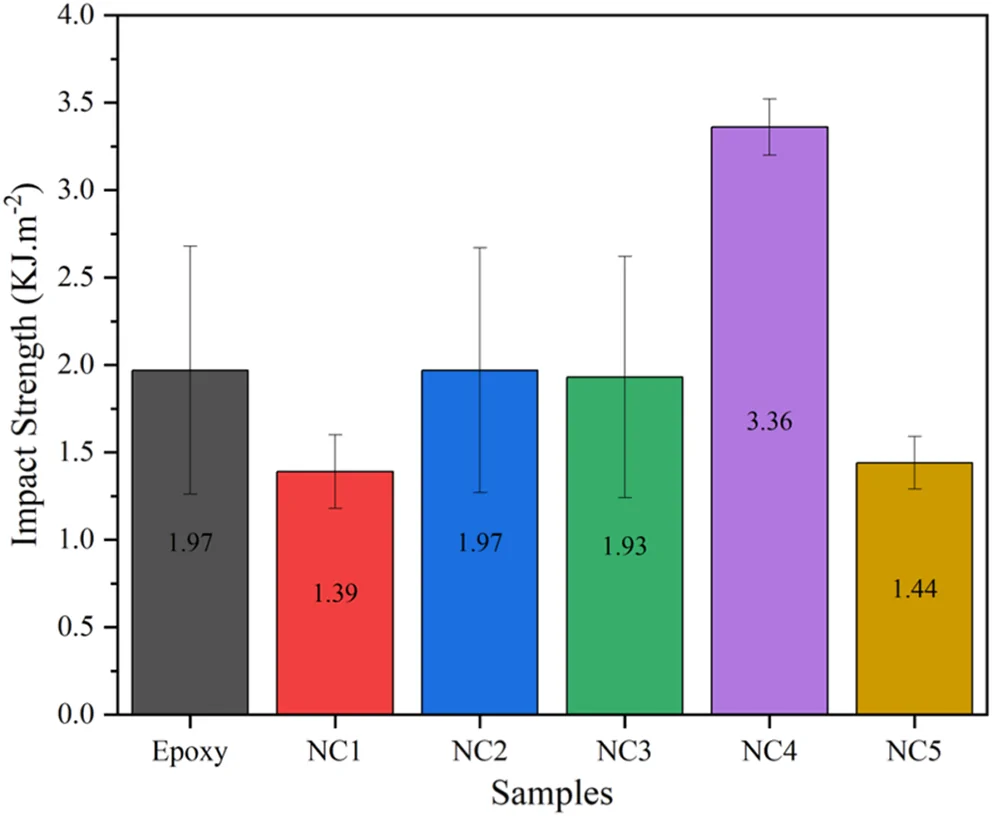
Impact test results of the pure resin and the nanocomposites.

**Figure 12:**
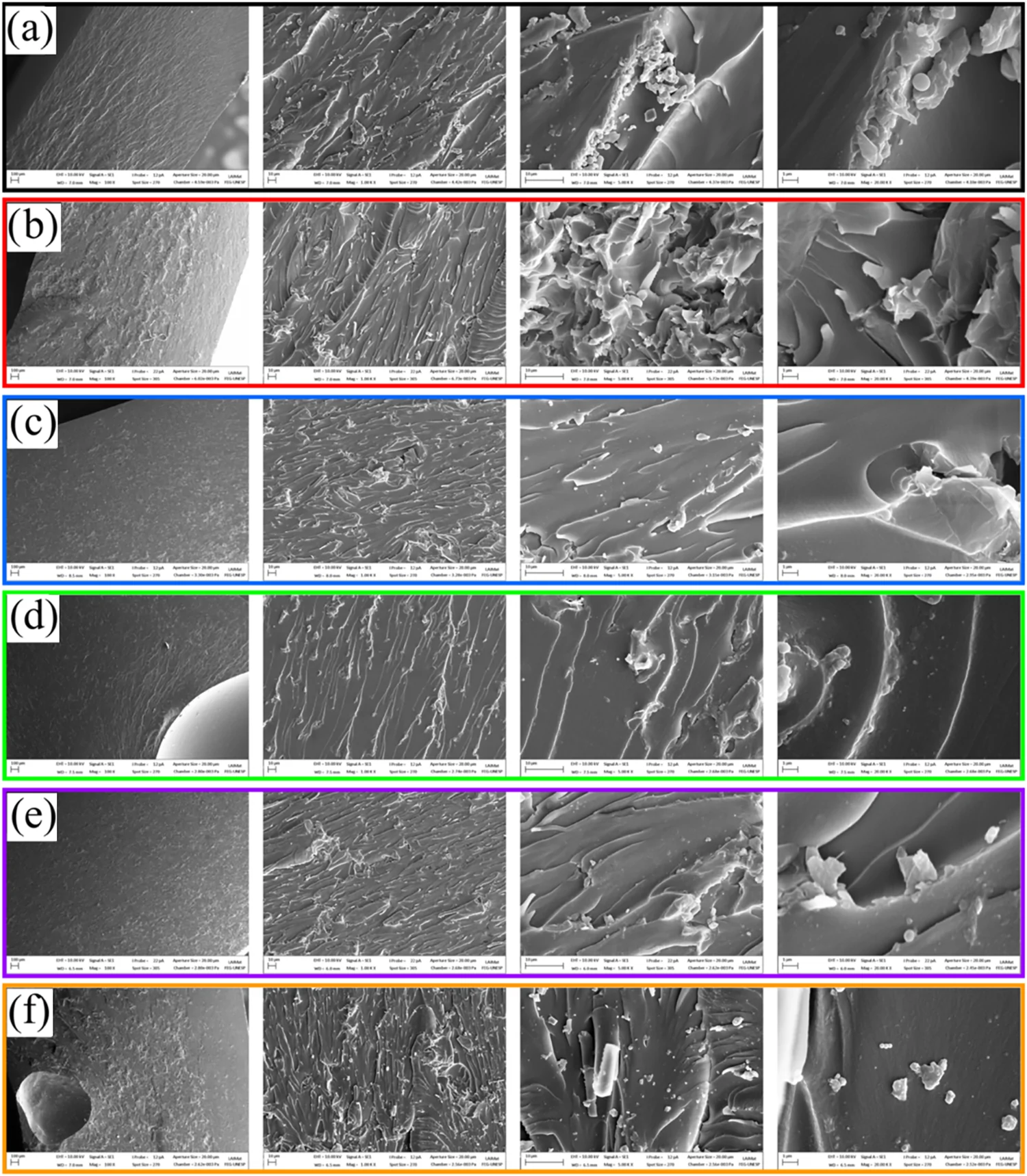
SEM micrographs of the fracture surfaces of neat epoxy resin and epoxy/GO nanocomposites after impact testing at magnifications of 100×, 1.000×, 5.000×, and 20.000×. The panels correspond to (a) neat epoxy and nanocomposites (b) NC1, (c) NC2, (d) NC3, (e) NC4, and (f) NC5 as indicated in the figure.

The results revealed that the neat epoxy resin exhibited an average value of (1.97 ± 0.71) kJ.m^–2^. The pure epoxy resin showed a relatively smooth fracture surface with a typical brittle fracture appearance, characterized by rapid crack propagation and low plastic deformation. This behavior is consistent with the impact resistance value obtained for the matrix, indicating a limited capacity for energy absorption prior to failure.^51,52^

For nanocomposites NC1 and NC5, which showed a reduction in impact resistance (29% and 27%, respectively), the micrographs revealed more irregular surfaces, with the presence of GO agglomerates and regions of interfacial discontinuity. Interlamellar failures and regions of more abrupt fracture were observed, suggesting that the less efficient dispersion of the nanoreinforcement acted as a stress concentrator, promoting early crack nucleation and propagation. This behavior explains the lower toughness of these systems and their tendency toward embrittlement.^53,54^

In samples NC2 and NC3, whose impact values were close to those of the neat resin, the fracture surfaces exhibited an intermediate morphology, with the presence of mixed fracture mechanisms. Some surface roughness and evidence of crack deflection around GO nanosheets were observed, along with relatively flat regions, indicating that the dispersion of the nanoreinforcement was not sufficiently effective to significantly enhance energy dissipation, although it contributed to stress redistribution within the matrix.^51,53^

Sample NC4, in turn, showed the best performance, with (3.36 ± 0.16) kJ.m^–2^, representing a gain of approximately 70.56% compared to the epoxy sample. This sample stood out not only for its high value but also for its low variability. This behavior suggests efficient GO dispersion, or possibly a synergistic effect of improved alignment of GO nanofillers and the activation of energy dissipation mechanisms such as crack deflection and nanofiller pull-out, resulting in a significant improvement in impact resistance.^55^ The low variance in the NC4 data further supports the hypothesis that this concentration corresponds to a more homogeneous and well-optimized formulation in terms of reinforcement, corroborating previous results obtained by TEM and DMA regarding the efficiency of the ultrasonic tip dispersion method. The presence of more tortuous fracture surfaces and microcavities also suggests the occurrence of localized deformation mechanisms, which contribute to delaying crack propagation and increasing the material’s toughness.

Although an increase in stiffness is often associated with reduced impact strength in polymer composites, the behavior observed for NC4 suggests that the ultrasonic-tip dispersion promoted a more favorable balance between reinforcement and toughness. The improved dispersion of GO increased the storage modulus by enhancing interfacial interactions and stress transfer efficiency. At the same time, the more homogeneous distribution of GO nanosheets contributed to crack deflection, crack path tortuosity, localized plastic deformation, microcavity formation, and possible nanofiller pull-out during impact fracture. These mechanisms increase the energy required for crack propagation, explaining the simultaneous improvement in stiffness and impact resistance observed for NC4.

Overall, the SEM analysis (Figure 12) confirms the strong relationship between the quality of GO dispersion and the mechanical performance of the nanocomposites. Systems with poor dispersion exhibited predominantly brittle fracture and lower impact resistance, whereas the nanocomposite produced with better dispersion showed more complex fracture mechanisms, higher surface roughness, and greater energy dissipation capacity. These results reinforce that the efficiency of the matrix/nanoreinforcement interface is a key factor in enhancing toughness and the overall mechanical performance of epoxy/GO nanocomposites.^56^

### 3.8 Comparative Visualization: Properties vs Dispersion Methods

The overall comparison of the properties of the neat epoxy matrix and the nanocomposites, according to the dispersion methods, was conducted using a radar chart, as illustrated in Figure 13. The analyzed variables include mechanical and dynamic-mechanical properties, such as storage modulus (*E*′), loss modulus (*E*″), impact resistance, glass transition temperature (*T*_g_), in addition to other important factors for nanostructured materials (*C*, *N*, *r*, *A*, and *b*), whose specific definitions have been discussed in detail earlier.

**Figure 13:**
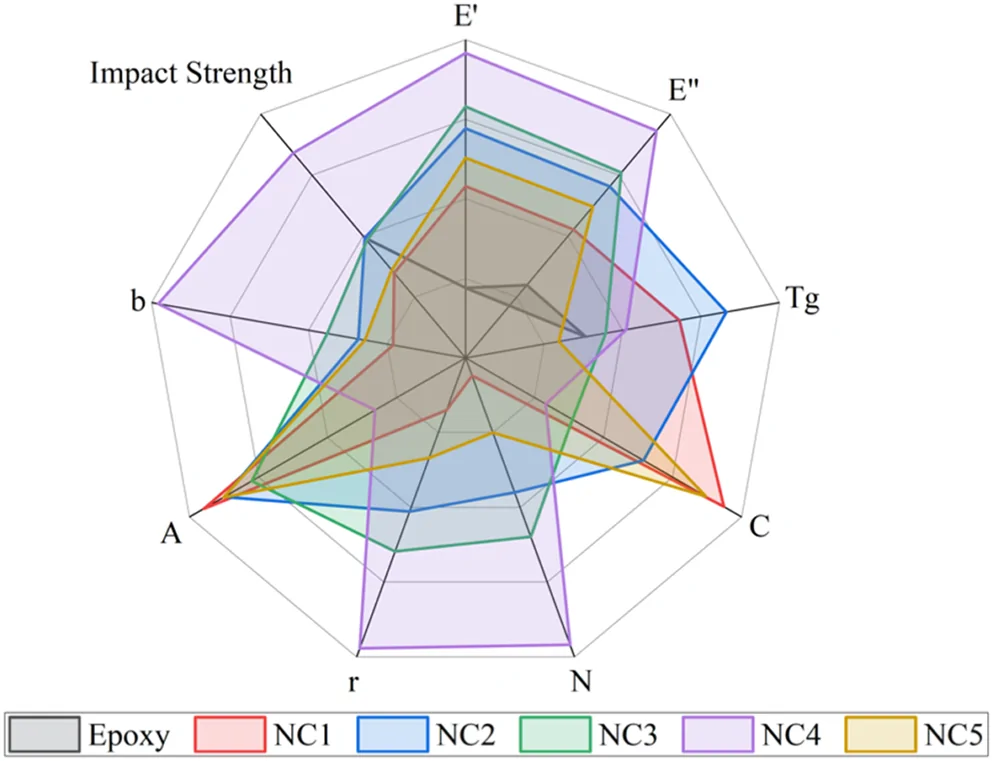
Comparative multiparameter evaluation of dispersion effectiveness: integrates the principal morphological, dynamic-mechanical and mechanical parameters into a single comparative representation.

Among the evaluated systems, the incorporation of GO significantly improved all properties, regardless of the dispersion method used. The improvements observed in storage modulus and impact resistance are particularly relevant for high-performance epoxy systems, where simultaneous stiffness and damage tolerance are desirable. Similar property enhancements have motivated the incorporation of graphene-based nanomaterials into epoxy matrices for potential aerospace applications, including lightweight structural components and adhesive systems.^57^ The NC4 nanocomposite stood out by presenting the largest total filled area in the radar chart, indicating superior and more balanced performance across nearly all analyzed variables. NC4 exhibited higher values of *E*′, *E*″, impact resistance, and more satisfactory parameter values, suggesting a more efficient synergy between the matrix and the nanofiller achieved through dispersion method. These results reinforce the positive effect of nanofiller incorporation on enhancing the properties of the epoxy system, with particular emphasis on the outstanding performance of NC4 composite.

In contrast, the results observed for NC1 are directly related to the technique used for its preparation. In this case, GO was incorporated into the resin by grinding of the nanofiller into the resin using a mortar and pestle, a process that relies solely on the operator’s applied force. This technique is ineffective at overcoming the van der Waals interactions between nanosheets, often leading to poor dispersion and the formation of agglomerates, justifying the results observed in this study. The low efficiency of this method is attributed to its inability to deliver sufficient energy for effective exfoliation of the material.

The technique used in NC2 provided greater shear compared to manual dispersion. However, its efficiency remained limited, especially due to the resin’s viscosity, which hinders the breakdown of larger agglomerates. The rotational movement of the impeller contributes to the distribution of the material but does not significantly exfoliate the GO nanosheets.

NC3 showed slightly improved results compared to NC2, likely due to a more homogeneous distribution of GO at moderate concentrations. The continuous rotation of the magnetic stirrer at the bottom of the container, combined with the friction generated, enhances surface dispersion.

NC4 exhibited superior performance compared to all other techniques. This method employs high-intensity ultrasonic waves directly in the resin, promoting cavitation and the microbubbles implosion that generate localized forces capable of breaking up agglomerates and exfoliating GO nanosheets. The result was a highly homogeneous dispersion, with no visible agglomerates and a greater degree of separation between the sheets, as illustrated in Figure 13.

Although NC5 also uses ultrasonic waves, the energy is distributed more diffusely in the liquid, resulting in less intense cavitation. Nevertheless, this method was able to promote a more efficient dispersion than the manual method.

Although the present study does not quantify the specific energy consumption of each dispersion method, the observed results indicate that dispersion efficiency depends not only on the total energy supplied but also on how this energy is transferred to the graphene oxide/epoxy system. In particular, ultrasonic tip dispersion concentrates acoustic energy in a localized region, generating intense cavitation that promotes effective exfoliation and homogeneous nanofiller distribution. Although ultrasonic tip dispersion provided the best performance under laboratory conditions, scaling this process to large composite structures presents several challenges. As resin volume increases, maintaining homogeneous graphene oxide dispersion becomes progressively more difficult due to higher viscosity, longer mixing paths, and reduced ultrasonic energy distribution throughout the material. In addition, temperature control, prevention of nanofiller reagglomeration, efficient degassing, and process reproducibility become increasingly important. Consequently, industrial implementation may require higher-power ultrasonic equipment, multiple sonication sources, or continuous-flow processing systems to achieve dispersion quality comparable to that obtained at laboratory scale.

Therefore, the selection of a dispersion technique for industrial applications should consider not only the resulting nanocomposite properties but also process scalability, energy efficiency, manufacturing cost, and production throughput.

From a practical perspective, the cost-performance balance of each dispersion method should also be considered (Table 3). Manual mixing is the simplest and lowest-cost approach, but it resulted in the lowest performance improvement among the GO/epoxy nanocomposites. Mechanical and magnetic stirring showed intermediate behavior, combining relatively simple processing with relevant increases in storage modulus and impact performance. The ultrasonic bath method requires higher equipment and energy input, but its performance was lower than that obtained with ultrasonic tip dispersion, suggesting less efficient energy transfer to the viscous epoxy/GO system.

**Table 3:** Qualitative Cost-Performance Assessment

Method	Sample	Processing cost	Performance	Cost-performance interpretation
Manual mixing	NC1	Very low	Moderate	Lowest cost, limited performance
Mechanical stirring	NC2	Low -moderate	High	Good balance
Magnetic stirring	NC3	Low–moderate	High	Good balance
Ultrasonic tip	NC4	High	Very high	Best performance, higher cost
Ultrasonic bath	NC5	Moderate–high	Intermediate	Lower efficiency than ultrasonic tip

Ultrasonic tip dispersion provided the highest performance, particularly for NC4, which exhibited the highest storage modulus, improved interfacial parameters, larger apparent interphase thickness, and enhanced impact resistance. However, this method requires more expensive equipment, higher local energy input, temperature control, and greater attention to processing scalability. Therefore, NC4 represents the best performance-oriented route, whereas mechanical stirring may offer a more favorable cost-performance compromise when processing simplicity and lower operational cost are prioritized.

As with most thermoset epoxy systems, the cross-linked network structure limits conventional mechanical recycling routes. Potential end-of-life strategies include thermal recovery, chemical recycling, and emerging reprocessing technologies developed for advanced thermoset systems. Considering the low GO concentration used in this work (0.5 wt %), the overall end-of-life behavior is expected to be primarily governed by the epoxy matrix.^58,59^

## 4 Conclusion

This study demonstrated that the direct solvent-free dispersion of graphene oxide (GO) into epoxy resin is a viable strategy for producing nanocomposites with improved thermal, dynamic-mechanical, and impact performance. All investigated dispersion methods improved the thermal, dynamic-mechanical, and impact properties of the neat epoxy resin to some extent. Among them, ultrasonic tip dispersion (NC4) yielded the most pronounced enhancements, achieving a 70.6% increase in impact resistance, a 157% improvement in storage modulus, and superior glass transition temperature performance. These results demonstrate that ultrasonic tip dispersion is the most effective method for promoting a homogeneous distribution of graphene oxide within the epoxy matrix, thereby enhancing interfacial adhesion and stress-transfer efficiency.

Mechanical stirring (NC2) and magnetic stirring (NC3) also provided significant improvements, representing viable alternatives for small- and large-scale production where high-energy ultrasonic equipment may not be available. In contrast, manual dispersion and ultrasonic bath (NC1 and NC5) were less effective, resulting in lower reinforcement efficiency and weaker interfacial interactions.

The present findings are applicable to the investigated processing conditions, including the graphene oxide content of 0.5 wt % and the solvent-free direct dispersion approach employed. Although the ultrasonic tip method exhibited the best overall performance, the present study was limited to a single graphene oxide concentration and did not directly quantify the effective ultrasonic energy transferred to the epoxy/GO system or possible chemical modifications of GO during sonication. Furthermore, no solvent-assisted dispersion methods were investigated, preventing a direct comparison between solvent-free and solvent-based processing routes. Future studies should evaluate different GO contents, surface functionalization strategies, hybrid nanofillers, and long-term durability under thermal cycling, fatigue, and environmental exposure, rheological and dielectric characterization to further expand the applicability of GO-reinforced epoxy nanocomposites. Such analyses may provide further insight into dispersion quality, interfacial interactions, network formation, and possible percolation phenomena within the epoxy matrix.

Beyond identifying ultrasonic tip processing as the most effective dispersion method under the investigated conditions, this work establishes a systematic relationship between the processing route, graphene oxide dispersion quality, interfacial behavior, and the resulting thermomechanical and impact performance of epoxy nanocomposites. Overall, this study highlights the importance of processing methodology as a key factor governing the structure–property relationships of epoxy/graphene oxide nanocomposites and provide practical guidance for the development of high-performance solvent-free nanocomposites for structural engineering applications.
